# A Hybrid Electrode of Co_3_O_4_@PPy Core/Shell Nanosheet Arrays for High-Performance Supercapacitors

**DOI:** 10.1007/s40820-015-0069-x

**Published:** 2015-10-15

**Authors:** Xiaojun Yang, Kaibing Xu, Rujia Zou, Junqing Hu

**Affiliations:** State Key Laboratory for Modification of Chemical Fibers and Polymer Materials, College of Materials Science and Engineering, Donghua University, Shanghai, 201620 People’s Republic of China

**Keywords:** Co_3_O_4_@PPy, Core/shell nanosheet arrays, Supercapacitors

## Abstract

Herein, combining solverthermal route and electrodeposition, we grew unique hybrid nanosheet arrays consisting of Co_3_O_4_ nanosheet as a core, PPy as a shell. Benefiting from the PPy as conducting polymer improving an electron transport rate as well as synergistic effects from such a core/shell structure, a hybrid electrode made of the Co_3_O_4_@PPy core/shell nanosheet arrays exhibits a large areal capacitance of 2.11 F cm^−2^ at the current density of 2 mA cm^−2^, a ~4-fold enhancement compared with the pristine Co_3_O_4_ electrode; furthermore, this hybrid electrode also displays good rate capability (~65 % retention of the initial capacitance from 2 to 20 mA cm^−2^) and superior cycling performance (~85.5 % capacitance retention after 5000 cycles). In addition, the equivalent series resistance value of the Co_3_O_4_@PPy hybrid electrode (0.238 Ω) is significantly lower than that of the pristine Co_3_O_4_ electrode (0.319 Ω). These results imply that the Co_3_O_4_@PPy hybrid composites have a potential for fabricating next-generation energy storage and conversion devices.

**Electronic supplementary material:**

The online version of this article (doi:10.1007/s40820-015-0069-x) contains supplementary material, which is available to authorized users.

## Introduction

With the rapid increasing demand in energy storage system for portable electronics and hybrid electric vehicles, supercapacitors have aroused widespread research interest owning to their high power density, fast charge–discharge rate and long lifespan [1–3]. As for a key component of the supercapacitors, electrode materials can be divided into three major types: carbon materials [4, 5], transition metal oxides [6–8] and conducting polymers (CPs) [9, 10]. Carbon materials store charges electrostatically through reversible ion adsorption at the electrode/electrolyte interface [11]. In comparison, transition metal oxides and CPs exploit the fast and reversible Faradic redox process at the electrode surface, thus delivering a considerably high specific capacitance [12, 13]. Therefore, the electrode materials based on transition metal oxides and CPs are gradually becoming a research hotspot in the field of the supercapacitors [14–16].

Among various electrode materials, Co_3_O_4_ is one of the most extensively investigated pseudocapacitive materials because of its low cost, environmental friendliness and high theoretical capacitance (~3560 F g^−1^) [8]. Importantly, it can provide multiple oxide states for reversible redox process [17]. Despite these appealing features, the real specific capacitance obtained from various Co_3_O_4_ nanostructures [18–20] is still far below the theoretical value, which may be attributed to its intrinsic semiconducting characteristic [21]. To overcome this problem, one effective method is fabricating addictive/binder-free electrode configuration to avoid the “dead surface” and tedious process in traditional slurry-coating electrode. Ni foam is widely used as the substrate to support metal oxides materials because of its good electrical conductivity and porous structure, which can enhance the electron transport and improve the active site of electrode materials. Simultaneously, another feasible method is designing three-dimensional (3D) hybrid electrode with large surface area and fast electron transport. Recently, integrating carbon materials, CPs, or noble metal nanoparticles onto electroactive materials has been demonstrated to be an effective synthesis route. Wang et al. [22] successfully prepared Co_3_O_4_@MWCNTs hybrid composites, which show superior electrochemical performance as positive electrode materials. As one of the most important CPs, polypyrrole (PPy) has been a promising pseudocapacitive electrode material because of its low cost, good electrical conductivity, relatively high capacitance, and outstanding mechanical flexibility [23]. For instance, Liu et al. [24] fabricated a supercapacitor electrode composed of CoO@PPy hybrid nanowires, which delivers a remarkably large areal capacitance of 4.43 F cm^−2^ at 1 mA cm^−2^, excellent rate capability and cycling performance; Hong et al. [25] developed a Co_3_O_4_@Au-PPy core/shell nanowires electrode, which exhibits a high specific capacitance of 2062 F g^−1^ (6.39 F cm^−2^) at 5 mA cm^−2^, with ~68 % retention of the initial capacitance from 5 to 50 mA cm^−2^. However, Au as a noble metal is quite costly, and the in situ interfacial polymerization process is time-consuming. In contrast, electrodeposition technique has great advantages, such as convenient, low cost, controllable, and efficient. Thus, it is of great interest to develop a low cost and efficient route to fabricate 3D Co_3_O_4_@PPy hybrid electrode with enhanced electrical conductivity and excellent electrochemical performance for supercapacitor applications.

Based on above consideration, we designed a 3D core/shell nanostructure of uniform PPy thin layer on mesoporous Co_3_O_4_ nanosheet arrays as a hybrid electrode material through a solvothermal and electrodeposition process. A hybrid electrode made of as-grown Co_3_O_4_@PPy core/shell nanosheet arrays exhibits a large areal capacitance of 2.11 F cm^−2^ at the current density of 2 mA cm^−2^, which is superior to 0.54 F cm^−2^ of the pristine Co_3_O_4_ electrode. Meanwhile, this electrode also displays a good rate capability (1.37 F cm^−2^ at the current density of 20 mA cm^−2^). Most importantly, the Co_3_O_4_@PPy hybrid electrode demonstrates a superior cycling performance (~85.5 % capacitance retention after 5000 cycles). Furthermore, the equivalent series resistance (ESR) value of the Co_3_O_4_@PPy hybrid electrode (0.238 Ω) is significantly lower than that of the pristine Co_3_O_4_ electrode (0.319 Ω), indicting the enhanced electrical conductivtity.

## Experimental

### Synthesis of Mesoporous Co_3_O_4_ Nanosheet Arrays

All reagents used in the work were of analytical grade. A hybrid electrode configuration was prepared by a facile two-step method, which can be easily scaled up. Typically, a piece of Ni foam (ca. 4 × 1 cm^2^) was carefully pretreated with 3 M HCl aqueous by ultrasonication for 30 min, and then cleaned with deionized water and absolute ethanol for several times. 2 mmol of Co(NO_3_)_2_·6H_2_O and 5 mmol of hexamethylenetetramine (HMT) were dissolved in 25 mL of deionized water and 25 mL of absolute ethanol under magnetic stirring for 30 min. Then, the resulting solution was transferred into a 60 mL Teflon-lined autoclave and a piece of cleaned Ni foam substrate was immersed into it. Subsequently, the autoclave was sealed and maintained in an electric oven at 90 °C for 8 h. After cooling down to room temperature naturally, the products were rinsed with deionized water and absolute ethanol for several times, and then dried at 60 °C for 2 h. Finally, the as-prepared samples were calcined at 300 °C in air for 2 h.

### Synthesis of Co_3_O_4_@PPy Core/Shell Nanosheet Arrays

PPy thin layer was grown on the surface of mesoporous Co_3_O_4_ nanosheet arrays by electrodeposition. The procedure of eletrodeposition was accomplished in a three-electrode system by using the Ni foam-supported as-grown Co_3_O_4_ electrode materials as the working electrode, a Pt foil as the counter electrode, and Ag/AgCl as the reference electrode. Electrolyte for electrodeposition of PPy was prepared by dissolving 0.4 mL of pyrrole (288 mM) and 0.1491 g of KCl (100 mM) into 20 mL of deionized water. Then, the Co_3_O_4_@PPy core/shell nanosheet arrays were synthesized at 0.8 V for a different duration of 2, 5, 8, and 10 min. Finally, as-prepared Co_3_O_4_@PPy hybrid electrode materials were rinsed with deionized water and absolute ethanol for several times, and then dried at 60 °C for 2 h.

### Structure Characterization

As-synthesized products were characterized by D/Max-2550 PC X-ray diffractometer (XRD, Rigaku, Cu-Kα radiation), X-ray photoelectron spectroscopy (XPS, PHI5000VersaProbe), scanning electron microscopy (SEM, HITACHI, S-4800) and transmission electron microscopy (TEM, JEOL, JEM-2100F) equipped with an energy-dispersive X-ray spectrometer (EDX). The Co_3_O_4_@PPy samples were easily scraped off from the Ni foam substrate for the Fourier transform infrared (FTIR) test, and the FTIR spectrum was recorded on a Nicolet 6700 FTIR spectrometer (Bruker).

### Electrochemical Characterization

Electrochemical measurements were performed on an Autolab electrochemical workstation (PGSTAT302N) using a three-electrode system and 1 M KOH as the electrolyte. A Pt foil and a saturated calomel electrode (SCE) were used as the counter electrode and the reference electrode, respectively. The Ni foam-supported Co_3_O_4_@PPy and Co_3_O_4_ electrode materials (ca. 1 cm^2^ area) acted directly as the working electrode.

## Results and Discussion

**Fig. 1:**
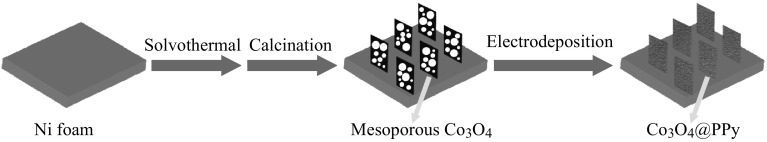
Schematic diagram for the synthesis of mesoporous Co_3_O_4_@PPy hybrid nanosheet arrays on Ni foam

In this study, the Co_3_O_4_@PPy hybrid nanosheet arrays were synthesized through a solvothermal and electrodeposition process. The synthesis procedure of the hybrid nanosheet arrays is briefly summarized in the accessible two steps as shown in Fig. 1. Firstly, mesoporous Co_3_O_4_ nanosheet arrays were grown vertically on the Ni foam via a solvothermal and calcination procedure. The 3D Ni foam has been widely employed as an ideal current collector owning to its uniform macropores, large supporting area (Fig. S1), and high electrical conductivity [26]. Secondly, PPy was continually integrated onto the surface of the mesoporous Co_3_O_4_ nanosheet arrays via a controllable and efficient electrodeposition technique. The detailed synthesis procedure of Co_3_O_4_@PPy hybrid nanosheet arrays was described in the Experimental section. In our design, the PPy shell not only enhances the electrical conductivity of the overall electrode that can facilitate electronic and ion diffusion and improve the utilization of electrode materials, but also contributes to the total capacitance owning to its synergistic effects. We envisage that such a unique hybrid nanostructured electrode together with abovementioned merits will display excellent electrochemical performance in charge storage.

**Fig. 2:**
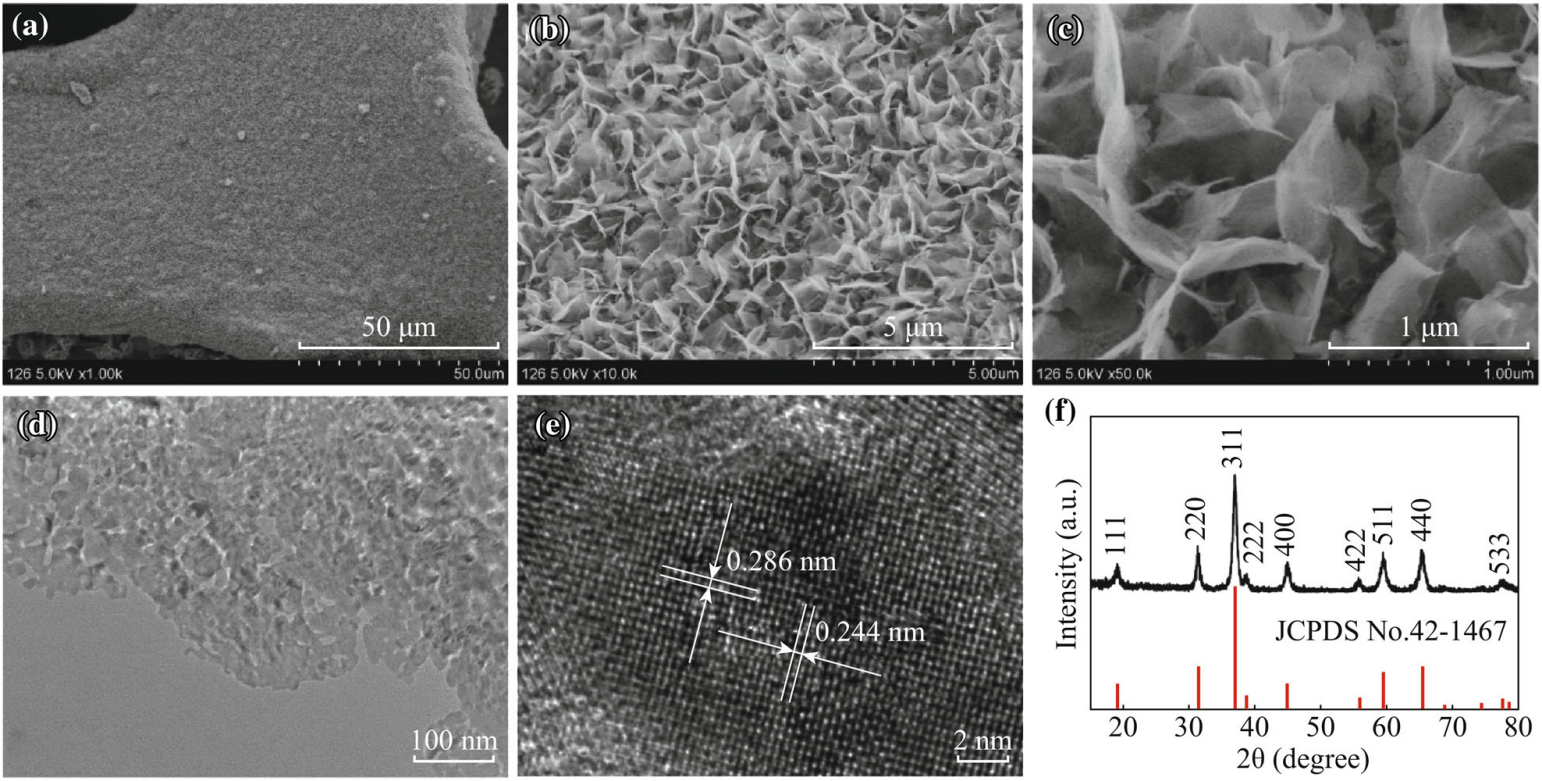
**a**–**c** Different magnification SEM images of the mesoporous Co_3_O_4_ nanosheet arrays on Ni foam. **d**, **e** TEM and HRTEM images of the Co_3_O_4_ nanosheets. **f** XRD pattern of the Co_3_O_4_ nanosheets scraped off from the Ni foam

Different magnification scanning electron microscopy (SEM) images of the pristine Co_3_O_4_ nanosheets are shown in Fig. 2a–c, respectively. A low-magnification SEM image (Fig. 2a) shows that the Co_3_O_4_ nanosheets are densely and uniformly grown on each strip of the Ni foam. As observed in higher-magnification SEM images (Fig. 2b, c), the Co_3_O_4_ nanosheets are interconnected with each other and approximately perpendicular to the Ni foam, forming a highly porous structure with broad open space. A TEM image (Fig. 2d) verifies that numerous mesopores are uniformly distributed throughout the overall surface of an individual Co_3_O_4_ nanosheet, and the porous size ranges from 2 to 10 nm, suggesting its mostly ultrathin feature. The formation of the mesopores could be related to the removal of water molecules during oxidative transformation of precursor to Co_3_O_4_ [27]. Such an electrode material with nearly vertical nanosheet arrays and highly porous structure can provide abundant electroactive sites, which is beneficial to charge transport and ion diffusion without the necessity of binder blocks, thereby resulting in improved charge transfer kinetics. A high-resolution TEM (HRTEM) image shown in Fig. 2e demonstrates that as-synthesized Co_3_O_4_ nanosheets give lattice fringes with interplanar spacings of 0.286 and 0.244 nm, corresponding to the (220) and (311) plane of the cubic Co_3_O_4_, respectively. The XRD pattern in Fig. 2f reveals the crystal structure and phase purity of as-synthesized Co_3_O_4_ nanosheets. All the diffraction peaks can be indexed into a pure face-centered cubic phase Co_3_O_4_ with a lattice constant of *a* = 8.08 Å (JCPDS Card No. 42-1467).

**Fig. 3:**
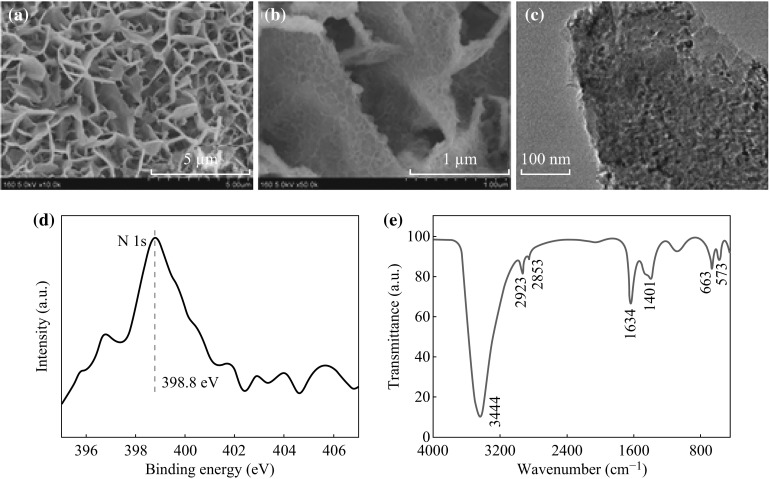
**a**–**c** SEM and TEM images of the Co_3_O_4_@ppy hybrid composites after 5 min electrodeposition. **d** XPS spectrum of N 1 s for the Co_3_O_4_@ppy hybrid composites. **e** FTIR adsorption spectrum of the Co_3_O_4_@ppy hybrid composites

The interconnected mesoporous Co_3_O_4_ nanosheet arrays can serve as an effective scaffold for loading additional electroactive pseudocapacitive electrode materials. In order to further enhance the electrochemical performance, PPy was chosen as an appropriate coating deposited on the mesoporous Co_3_O_4_ nanosheet arrays. The morphology and structure of the Co_3_O_4_@PPy hybrid composites were characterized by SEM and TEM. As shown in Fig. 3a, b, wrinkle-like PPy thin layer densely covers the surface of the Co_3_O_4_ nanosheets. Notably, the decoration of the PPy coating significantly increases the thickness and surface roughness of the Co_3_O_4_ nanosheets, whereas the Co_3_O_4_@PPy hybrid composites well maintain the ordered nanostructure. The TEM image in Fig. 3c clearly illustrates that partial porous structure has been covered by the PPy coating, as compared with the pristine Co_3_O_4_ nanosheets. Moreover, XPS measurement was employed to prove the existence of the PPy coating. The binding energy of N 1 s peak (Fig. 3d) is centered at 398.8 eV, which corresponds to the neutral nitrogen moieties (–NH–) on PPy [28, 29]. Figure 3e shows the FTIR adsorption spectrum of the Co_3_O_4_@PPy hybrid composites. A strong adsorption peak at 3444 cm^−1^ should be the stretching vibration of N–H. Two peaks at 1634 and 1401 cm^−1^ are induced by C=C and C–N on the pyrrole ring, respectively [30]. The peaks at 2923 and 2853 cm^−1^ are designated as the asymmetric stretching and symmetric vibrations of CH_2_ [31]. Other obvious peaks at 663 and 573 cm^−1^ are attributed to Co–O stretching in Co_3_O_4_ [32]. According to abovementioned characterizations, we convince that the Co_3_O_4_@PPy hybrid composites have been successfully synthesized.

**Fig. 4:**
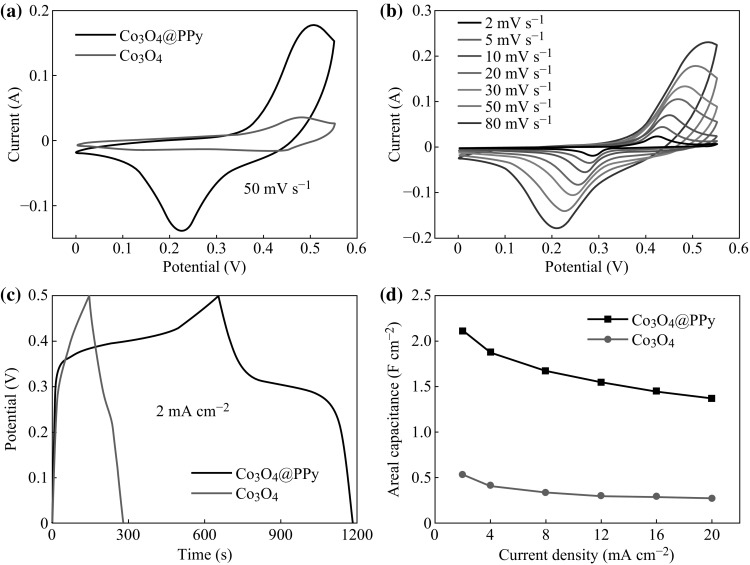
**a** CV curves of the Co_3_O_4_@ppy hybrid electrode and Co_3_O_4_ electrode at a scan rate of 50 mV s^−1^. **b** CV curves of the Co_3_O_4_@ppy hybrid electrode and Co_3_O_4_ electrode at various scan rates. **c** CD curves of the Co_3_O_4_@ppy hybrid electrode and Co_3_O_4_ electrode with a current density of 2 mA cm^−2^. **d** Areal capacitances of the Co_3_O_4_@ppy hybrid electrode and Co_3_O_4_ electrode at various current densities

To evaluate the electrochemical performance of the Co_3_O_4_@PPy hybrid electrode using the Co_3_O_4_ electrode as a comparison, electrochemical measurements were conducted in a three-electrode cell with a Pt counter electrode and a SCE reference electrode in 1 M KOH electrolyte. Figure 4a shows the cyclic voltammetry (CV) curves of the Co_3_O_4_@PPy hybrid electrode and Co_3_O_4_ electrode at a scan rate of 50 mV s^−1^ with the potential range of 0 to 0.55 V. It is particularly noteworthy that after coating a PPy thin layer, the enclosed CV curve of the Co_3_O_4_@PPy hybrid electrode expands drastically, indicating that much larger capacitance is obtained owning to their synergistic effects from two materials of Co_3_O_4_ and PPy. Firstly, PPy can provide good electrical conductivity, which will definitely result in improved electron transport rate through individual nanosheets. Secondly, PPy itself behaves additional pseudocapacitance during ion doping/dedoping in alkaline solution [24]. Figure 4b displays the CV curves of the Co_3_O_4_@PPy hybrid electrode at various scan rates. The profile of these curves is not significantly changed with an increasing scan rate from 2 to 80 mV s^−1^, indicating a reversible electrochemical process and an ideal pseudocapacitive characteristic. In addition, the redox peaks slowly move toward positive/negative potential along with the increasing of scan rate, revealing a good contact between the electroactive Co_3_O_4_@PPy nanosheets and the conductive Ni foam substrate. Figure 4c shows the galvanostatic charge–discharge (CD) curves of the Co_3_O_4_@PPy hybrid electrode and Co_3_O_4_ electrode at the current density of 2 mA cm^−2^. As expected, the Co_3_O_4_@PPy hybrid electrode displays much longer discharging time than the pristine Co_3_O_4_ electrode. It denotes that the Co_3_O_4_@PPy hybrid electrode exhibit much larger areal capacitance than the pristine Co_3_O_4_ electrode, corresponding to the CV test. The areal capacitances of the Co_3_O_4_@PPy hybrid electrode and Co_3_O_4_ electrode are calculated based on the discharge curves (Fig. S2) measured at various current densities via the following formula [33]: *C* = *I*Δ*t*/*S*Δ*V*, where *I* (A) is the discharge current, Δ*t* (s) is the discharge time, *S* is the geometric area of the active electrode, and Δ*V* (V) is the voltage interval, as illustrated in Fig. 4d. Correspondingly, the areal capacitances of the Co_3_O_4_@PPy hybrid electrode with different electrodeposition times are examined and plotted in Fig. S3. The Co_3_O_4_@PPy hybrid electrode after 8 min electrodeposition delivers the largest areal capacitance of 2.11 F cm^−2^ at the current density of 2 mA cm^−2^, which is remarkably larger than the value obtained for the pristine Co_3_O_4_ electrode (0.54 F cm^−2^). The Co_3_O_4_@PPy hybrid electrode still has an areal capacitance of 1.37 F cm^−2^ when the current density is increased to 20 mA cm^−2^, demonstrating its outstanding rate capability (~65 %). For comparison, the capacity retention of the pristine Co_3_O_4_ electrode is only ~50 % at the current density of 20 mA cm^−2^. To our best knowledge, the excellent electrochemical performance of the Co_3_O_4_@PPy hybrid electrode presented here is superior to those of previously reported electrodes (see Table 1). Such a large areal capacitance of the as-synthesized Co_3_O_4_@PPy hybrid electrode will demonstrate a great advantage in improving the energy density of supercapacitors.

**Table 1:** Comparison of performance metrics for the Co_3_O_4_@PPy electrode materials with several reported electrode materials in previous literatures

Electrode materials	Areal capacitance	Refs.
Co_3_O_4_@PPy hybrid composites	2.11 F cm^−2^ at 2 mA cm^−2^	This work
Mesoporous Co_3_O_4_ nanosheets	0.54 F cm^−2^ at 2 mA cm^−2^	This work
Co_3_O_4_@PPy@MnO_2_ core/shell/shell nanowires	1.13 F cm^−2^ at 1.2 mA cm^−2^	[34]
Co_3_O_4_@PPy@MnO_2_ ternary core/shell composites	0.55 F cm^−2^ at 0.5 A g^−1^	[35]
Co_3_O_4_@MnO_2_ core/shell nanowires	0.56 F cm^−2^ at 11.25 mA cm^−2^	[6]
Co_3_O_4_@NiO core/shell nanowires	1.35 F cm^−2^ at 6 mA cm^−2^	[12]
ZnO@MnO_2_@PPy ternary core/shell nanorods	1.793 F cm^−2^ at 2 A g^−1^	[36]
FEG/PPy hybrid composites	0.56 F cm^−2^ at 1 mA cm^−2^	[37]

**Fig. 5:**
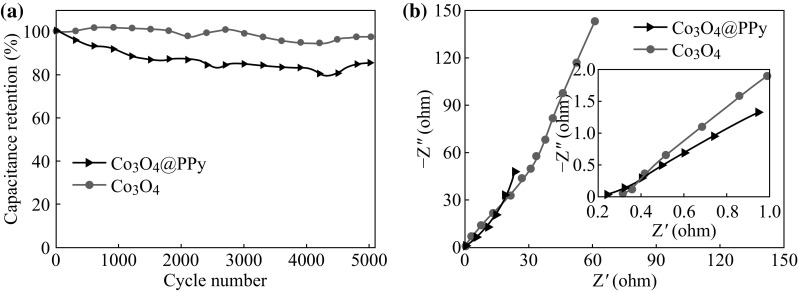
**a** Cycling performance of the Co_3_O_4_@ppy hybrid electrode and Co_3_O_4_ electrode tested at a scan rate of 50 mV s^−1^ for 5000 cycles. **b** Compared EIS curves of the Co_3_O_4_@ppy hybrid electrode and Co_3_O_4_ electrode. The *inset* delivers the enlarged nyquist plots at higher frequency

Cycling performance is another key factor for supercapacitor applications. Herein, a long-term cycling performance of the as-synthesized electrode materials was examined and compared at a scan rate of 50 mV s^−1^ for 5000 cycles, as shown in Fig. 5a. The overall capacitance retention of the Co_3_O_4_@PPy hybrid electrode can reach ~85.5 % after 5000 cycles, indicting a superior cycling performance [38–41]; as a comparison, the overall capacitance retention of the pristine Co_3_O_4_ electrode is 97.7 %, suggesting that the Co_3_O_4_@PPy hybrid electrode has a ~12 % decrease of the capacitance retention. As is known to all, PPy intrinsically exhibits poor cycling performance caused by its large volumetric swelling and shrinking during ion doping/dedoping process [42]. Therefore, it is not difficult to understand the declining of cycling performance after coating the PPy thin layer. In order to further investigate the electrochemical performance of the as-synthesized electrode materials, electrochemical impedance spectroscopy (EIS) measurement was also conducted to evaluate the electrical conductivity and ion diffusion. As shown in Fig. 5b, the Nyquist plots at higher frequency deliver the ESR value of the Co_3_O_4_@PPy hybrid electrode (0.238 Ω) and the pristine Co_3_O_4_ electrode (0.319 Ω), indicating the enhanced electrical conductivity after coating the PPy thin layer [43]. The EIS results imply the easy penetration of the electrolyte into the hybrid electrode and the improved utilization rate of the electrode materials, which can well explain the significantly enhanced areal capacitance as discussed above.

## Conclusion

In summary, a hybrid nanomaterial of Co_3_O_4_@PPy core/shell nanosheet arrays on Ni foam was prepared through a solvothermal and electrodeposition process. The Co_3_O_4_@PPy hybrid electrode exhibits a large areal capacitance of 2.11 F cm^−2^ at the current density of 2 mA cm^−2^, a ~4-fold enhancement compared with the pristine Co_3_O_4_ electrode. Furthermore, the Co_3_O_4_@PPy hybrid electrode also displays good rate capability (~65 % retention of the initial capacitance from 2 to 20 mA cm^−2^) and superior cycling performance (~85.5 % capacitance retention after 5000 cycles). In addition, the ESR value of the Co_3_O_4_@PPy hybrid electrode (0.238 Ω) is significantly lower than that of the pristine Co_3_O_4_ electrode (0.319 Ω). The outstanding electrochemical performance can enable the Co_3_O_4_@PPy hybrid composites to be a promising electrode material for next-generation energy storage and conversion devices.

## Electronic supplementary material

Below is the link to the electronic supplementary material.

Supplementary material 1 (DOC 1771 kb)
