# Supplementary material for: A Hybrid Electrode of Co3O4@PPy Core/Shell Nanosheet Arrays for High-Performance Supercapacitors
Source: Nanomicro Lett. 2015 Oct 15;8(2):143–50. doi: 10.1007/s40820-015-0069-x (PMC6223670; doi:10.1007/s40820-015-0069-x)
Supplement: Supplementary file 1 — Supplementary material 1 (DOC 1771 kb) [file 40820_2015_69_MOESM1_ESM.doc]

Supporting Information for

**A Hybrid Electrode of Co_3_O_4_@PPy Core/Shell Nanosheet Arrays for High-performance Supercapacitors**

Xiaojun Yang, Kaibing Xu, Rujia Zou*, Junqing Hu*

State Key Laboratory for Modification of Chemical Fibers and Polymer Materials, College of Materials Science and Engineering, Donghua University, Shanghai 201620, People’s Republic of China

*Corresponding authors. E-mail: rjzou@dhu.edu.cn, hu.junqing@dhu.edu.cn

**Fig. S1** SEM image of the Ni foam

**Fig. S2** (**a, b**) CD curves of the Co_3_O_4_@PPy hybrid electrode and Co_3_O_4_ electrode at various current densities

**Fig. S3** (**a**) CD curves and (**b**) areal capacitance of the Co_3_O_4_@PPy hybrid electrode as a function of the PPy electrodeposition time


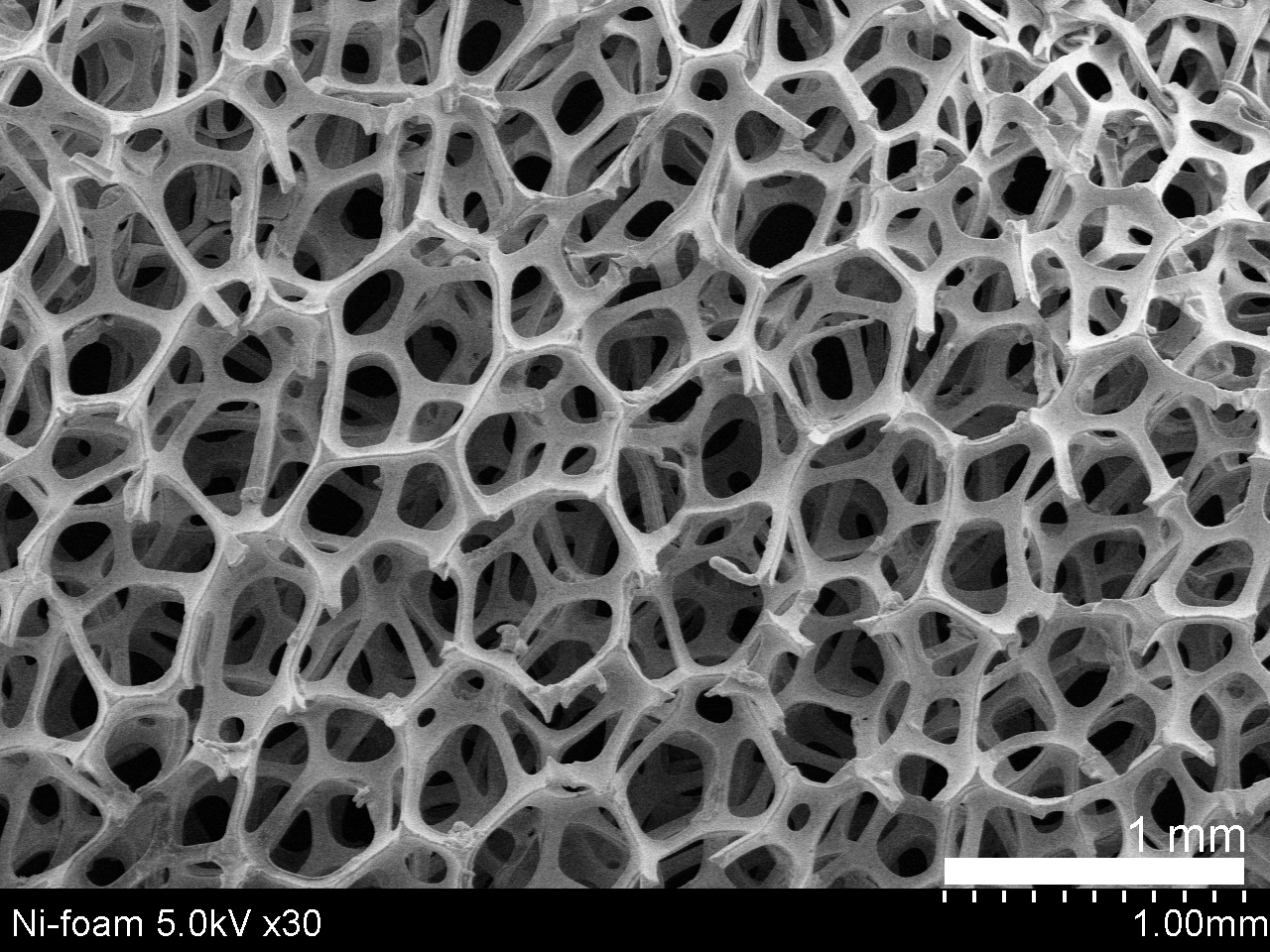


**Fig. S1**


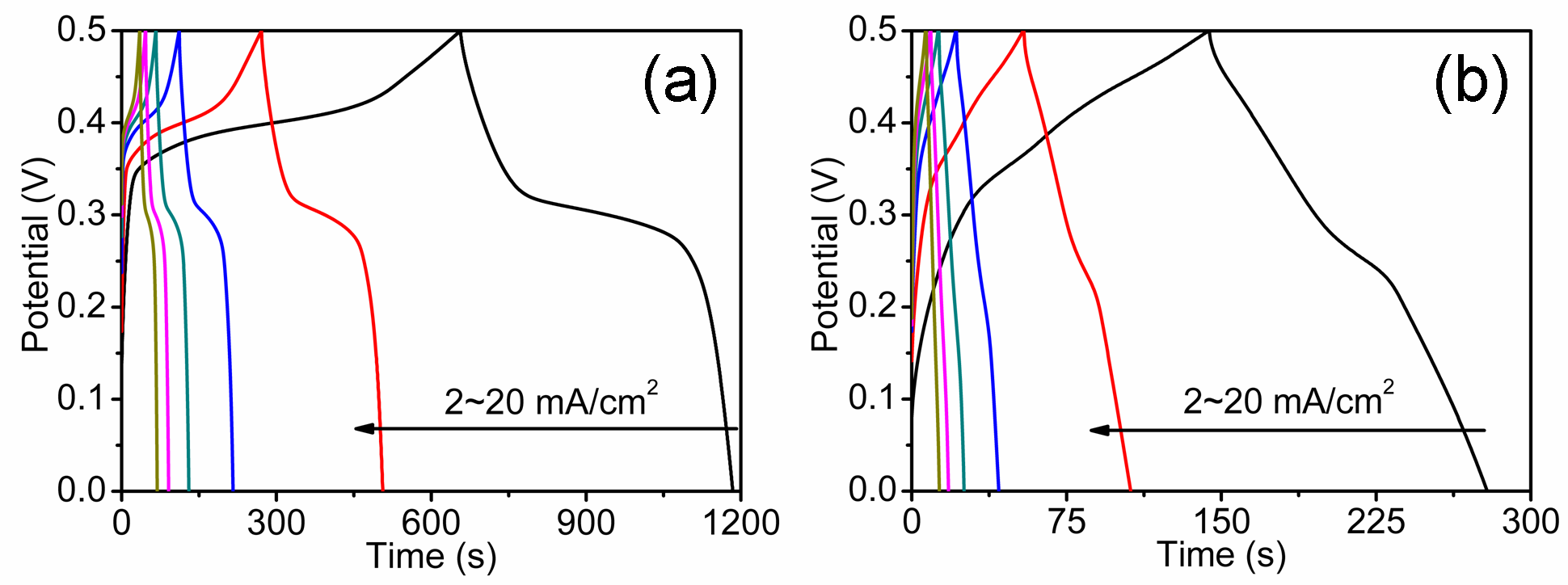


**Fig. S2**


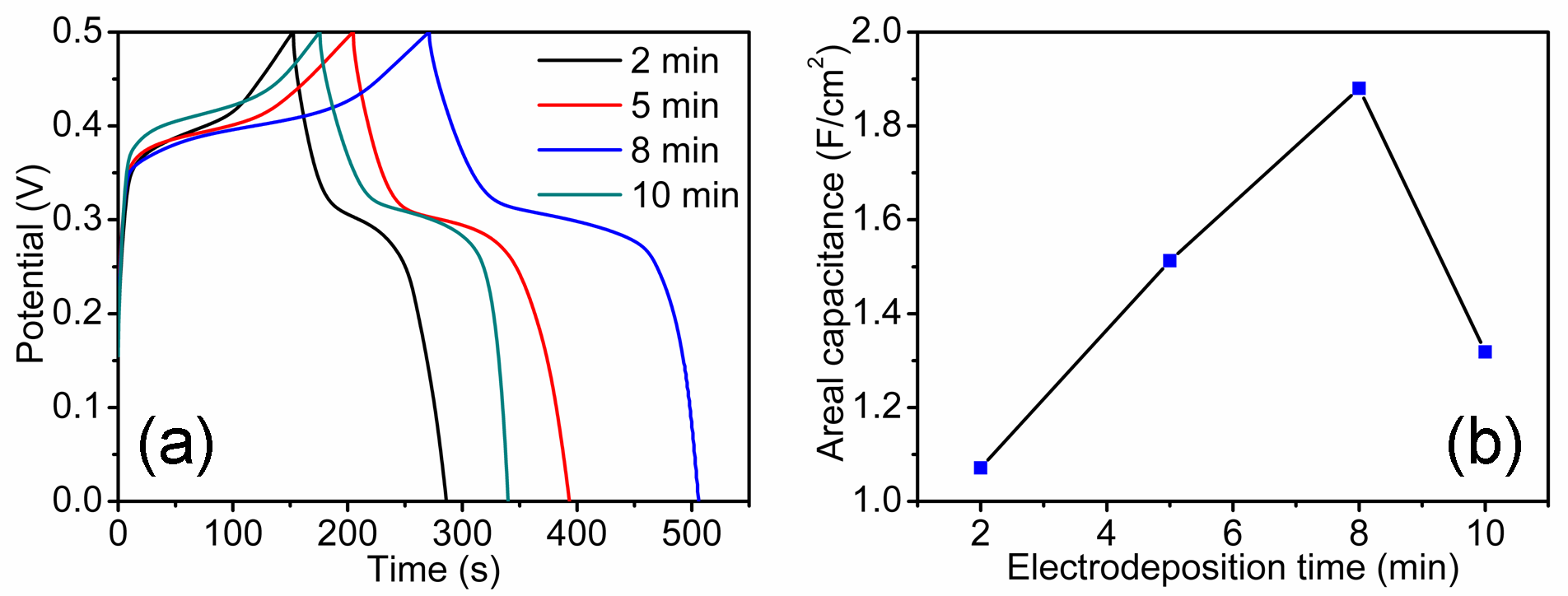


**Fig. S3**
